# A new cell line for high throughput HIV-specific antibody-dependent cellular cytotoxicity (ADCC) and cell-to-cell virus transmission studies

**DOI:** 10.1016/j.jim.2016.03.002

**Published:** 2016-06

**Authors:** Chiara Orlandi, Robin Flinko, George K. Lewis

**Affiliations:** The Institute of Human Virology of the University of Maryland School of Medicine, Baltimore, MD, USA

**Keywords:** RFADCC, EGFP-CEM-NKr-CCR5-SNAP, Flow-cytometry, Monoclonal antibody, HIV-1 virus

## Abstract

Several lines of evidence indicate that antibody-dependent cellular cytotoxicity (Wren et al., 2013) is important in the pathogenesis of HIV-1 infection. Namely, ADCC is induced during natural HIV-1 infection or in HIV-1 vaccine studies, the latter demonstrated by the RV144 vaccine trial. To expedite the assessment of ADCC in studies of HIV, we have developed a high throughput assay. We have optimized the rapid fluorometric antibody-mediated cytotoxicity assay (RFADCC) by transfecting the EGFP-CEM-NKr cell line to constitutively express SNAP-tagged CCR5. This cell line can then serve as a source of HIV-specific targets when coated with monomeric gp120, spinoculated with inactivated intact virions, infected by cell-free viral diffusion or infected by cell-to-cell transmission of virus. The optimized strategy has two significant advantages over the original RFADCC method: First, the preparation of detectable target cells is less labor intensive and faster as it does not rely on multiple staining and washing steps for target cells. Second, because the target cell markers GFP and SNAP are constitutively expressed, the assay provides highly reproducible data. These strengths make the optimized RFADCC assay suitable not only for studies of HIV-1 specific cytotoxicity but also for studies of cell–cell transmission of virus. In conclusion, this assay provides a new generation T cell line that can expedite large clinical studies as well as research studies in humans or non-human primates.

## 1 Introduction

ADCC in HIV-1 has been studied for over 20 years (Wren et al., 2013), but interest in the HIV-specific response was prompted by findings from the recent RV144 clinical vaccine trial showing ADCC, together with low IgA, as a correlate of protection (Bonsignori et al., 2012, Haynes et al., 2012). Also, observations obtained in several natural HIV infection systems (Chung et al., 2011, Ferrari et al., 2011) have highlighted a key role of ADCC activity in the immune response against the virus. A number of experimental assays have been standardized and utilized to characterize human or non-human primate antibodies for HIV-specific cytotoxicity. Many of the ADCC assays measure the potency of antibodies to mediate killing of virus-infected target T cells, mainly CEM NKr CCR5, by healthy, uninfected donor PBMC effector cells. These assays rely on the quantification of target cells that are pre-labeled with traceable compounds, the loss of which indicates a decrease in membrane integrity or decrease in target cell viability. As ADCC readouts, these assays exploit critical steps of cytotoxicity, such as release of ^51^chromium due to apoptotic killing of specific targets (Ahmad et al., 2001), release of granzyme B by activated effectors (Pollara et al., 2011), loss of intracellular carboxyfluorescein diacetate succinimidyl ester (CSFE) due to disruption of target cell membrane integrity (Gomez-Roman et al., 2006) or decrease in luciferase signal due to direct killing of virus-bearing luciferase targets (Liao et al., 2013, Pollara et al., 2014). In addition, a novel ADCC assay that incorporates a CD16^+^ NK effector cell line and a CD4^+^ T-cell line expressing HIV Tat-inducible luciferase (Alpert et al., 2012) has been utilized to identify the inverse correlation between ADCC titers and risk of infection in the RV144 trial (Bonsignori et al., 2012, Haynes et al., 2012). More recently, another ADCC assay based on the quantification of killed targets using the cell marker eFluor670 and a live/dead dye was reported (Richard et al., 2014). Although these assays have provided important information about HIV pathogenesis or design and delivery of HIV vaccines, most are typically labor intensive and time consuming. Likewise, the RFADCC assay employed by our group to characterize mAbs specific for highly conserved regions of HIV-1 envelope exposed during viral entry (Gomez-Roman et al., 2006, Guan et al., 2013, Acharya et al., 2014) is equally demanding. However, because flow cytometry analyses have allowed a detailed understanding of the phenotype of the cells involved, we modified the original RFADCC assay to streamline the manipulations and improve the inter-experimental reproducibility. To this end, we optimized our RFADCC assay to avoid the need for the cumbersome and multiple staining steps and washings, including eliminating the harsh target cell membrane staining with PKH26. The modified assay now involves only one rapid staining step and is highly useful for the systematic analysis of ADCC using target cells either sensitized with gp120, spinoculated with intact HIV virions, infected by cell-free virus or by cell-to-cell transmission of virus.

## 2 Material and methods

### 2.1 Cell lines, viruses and monoclonal antibodies (mAbs)

The human T lymphoblastoid cell lines CEM NKr CCR5 (Howell et al., 1985) and EGFP-CEM-NKr (Kantakamalakul et al., 2006) were obtained from the National Institute of Allergy and Infectious Diseases (NIAID) Reagent Repository. CEM NKr CCR5 cells were maintained in RPMI 1640 medium with glutamine and supplemented with 10% fetal bovine serum (FBS), HEPES buffer (Sigma) 10 mM, sodium pyruvate (Sigma) 500 μM, β-Mercaptoethanol (GIBCO) 50 μM and gentimicin (GIBCO) 50 μg/ml (termed R10 medium). The EGFP-CEM-NKr and EGFP-CEM-NKr-CCR5-SNAP cells were maintained in R10 medium supplemented with neomycin G418 (Gemini, Bio-Products) at 800 μg/ml and 1.5 mg/ml, respectively.

For cell-bound virion studies, we employed AT-2 inactivated HIV-1 BalSuPT1-CCR5 CL.30 at a multiplicity of infection (MOI) of 5. This MOI was calculated using analytical information provided by Dr. Jeffery Lifson (National Cancer Institute at Frederick, Frederick, MD), who generously supplied this preparation. For infection by cell-free virus or cell–cell virus spread, we used HIV-1 Bal infectious molecular clone (NIH AIDS Reagent & Reference Reagent Program Lot. 9 021056) (Gartner et al., 1986) and HIV-1 Bal infectious molecular clone produced in SupT1-R5 cells (Ray et al., 2014) respectively. The human mAbs included in the study were isolated from our Natural Virus Suppressors (NVS) cohort (Sajadi et al., 2007, Sajadi et al., 2009) and characterized to be specific for highly conserved regions of CD4-triggered gp120 (Guan et al., 2013): N5-I5 targets the mAb A32-like epitope surface around β$\overset{-}{2}$, β1$\overset{-}{11}$-strands and α0- and α1-helixes of layers 1 and 2 of the inner domain of gp120 (Moore et al., 1994, Finzi et al., 2010, Pancera et al., 2010, Acharya et al., 2014, Gohain et al., 2015); C11 targets a distinct and discontinuous epitope on the seven-stranded β-platform and a residue in the extended C terminus of the gp120 molecule (Moore et al., 1994, Pancera et al., 2010, Pollara et al., 2013, Gohain et al., 2015); N12-i12 targets the gp120 co-receptor binding site (CoRbs) (Guan et al., 2013); N10-U1 targets the immunodominant domain of gp41 (unpublished result). The anti-respiratory syncytial virus mAb Synagis (MedImmune) was used as a negative control in most experiments.

### 2.2 Production of EGFP-CEM-NKr cells stably expressing CCR5-SNAP

EGFP-CEM-NKr cells (2 × 10^6^) were transfected with 2 μg of Tag-lite pSNAP-CCR5 vector (pSNAPCCR5, *htfr*) with the Amaxa nucleofector and Amaxa Cell line Nucleofector Kit C (Cat. VCA-1004) according to the manufacturer's instructions. EGFP-CEM-NKr cells were maintained with R10 medium supplemented neomycin at 800 μg/ml during and after transfection due to the EGFP vector resistance. On day 2 post-transfection, the expression of CCR5-SNAP molecule on the cell surface was assessed by flow cytometry of cells stained with PE anti-CCR5 mAb 2D7 (BD Pharmingen Cat. 555993).

The CCR5-positive EGFP-CEM-NKr cells were subjected to three rounds of selection by labeling with the 2D7 mAb and sorting with a FACSARIA II (Becton Dickinson, BD). Finally, when it was determined that CCR5 expression peaked on EGFP-CEM-NKr CCR5-SNAP cells, clones that showed distinct high levels of CCR5 expression were selected by limiting dilution. The expression of the SNAP tag was also assessed by indirect staining with rabbit anti-SNAP-Tag pAb (GenScript Corporation Cat. A00684) and PE F(ab′)2 donkey anti-rabbit IgG (eBioscience Cat. 12-4739) (data not shown). EGFP-CEM-NKr-CCR5-SNAP cells were passaged twice per week, with a higher neomycin dose (1.5 mg/ml) in order to better induce the CCR5-SNAP molecule expression on the cell surface and monitored weekly by flow cytometry. Quantibrite PE beads (Phycoerythrin Fluorescence Quantification Kit, BD Bioscience) were used to estimate the number of CCR5 and CD4 molecules present per EGFP-CEM-NKr-CCR5-SNAP cell, with CEM NKr CCR5 cells used as a reference.

### 2.3 Optimized RFADCC assay and cell surface staining

To optimize the RFADCC assay (Gomez-Roman et al., 2006) for high throughput efficiency, the regular double staining with the membrane PKH-26 dye and the intracellular carboxyfluorescein diacetate, succinimidyl ester (CSFE) dye were replaced with the membrane staining of the CCR5-SNAP-tag and the constitutive intracellular expression of GFP. For the ADCC protocol (Fig. 2), EGFP-CEM-NKr-CCR5-SNAP target cells were stained with the fluorescent substrate SNAP-Surface Alexa Fluor 647 (New England BioLabs Cat. S9136S) for 20 min at 37 °C with or without coating of monomeric HIV-1 Bal gp120 (50 μg/ml). For the studies with spinoculated virus, the cells were first stained with the SNAP-Surface dye and then spinoculated with the AT-2 inactivated Bal HIV-1 virus at 2000 RPM for 2 h at 12 °C. Gp120-sensitized, virus-spinoculated or infected EGFP-CEM-NKr-CCR5-SNAP target cells were then washed twice with cold R10 medium and added to a 96-well V-bottom plate (5000 cells/well). A final volume of 100 μl/well of antibody dilution was added and incubated with sensitized targets for 15 min at room temperature. A total of 250,000 purified human effector PBMC isolated by Ficoll-Paque from the whole blood of healthy human donors cells were added to each well at an effector/target (E/T) ratio of 50:1. After incubation at 37 °C in 5% CO_2_ for 2 h (gp120-coated) or 3 h (virus-spinoculated or infected cells), cells were washed with phosphate-buffered saline (PBS) containing 1% FBS (wash buffer), and fixed in 1% paraformaldehyde. Samples were analyzed at approximately 35,000 events per sample on an LSRII Fortessa flow cytometer (BD Biosciences). Doublets were excluded by forward-scatter area (FSC-A) versus forward-scatter height (FSC-H). Data were analyzed by FlowJo software (Tree Star, Ashland, OR). ADCC activity (=% cytotoxicity) is defined as the percentage of EGFP-CEM-NKr-CCR5-SNAP target cells that lose GFP staining but retain the CCR5-SNAP tag dye.

For cell surface staining, HIV-1 gp120-sensitized and infected EGFP-CEM-NKr-CCR5-SNAP target cells were incubated for 30 min at RT with 1 and 5 μg/ml, respectively, of Alexa-Fluor 647-conjugated mAbs C11, N5-i5 or N12-i2 in PBS. Target cells spinoculated with Bal AT-2 inactivated virus were incubated for 30 min at RT, respectively with 2 μg/ml of Alexa-Fluor 594-conjugated mAbs C11, N5-i5, N12-i2 or N10U1 in PBS. Cells were then washed once with wash buffer and fixed in a 1% paraformaldehyde.

### 2.4 HIV-1 infection of EGFP-CEM-NKr-CCR5-SNAP cells

To prepare the EGFP-CEM-NKr-CCR5-SNAP cells for virus infection assays, the cells were split 1:2 the previous day. Spinoculation was performed in a 96-well U-bottom plate by resuspending 5 × 10^5^ target cells in a medium containing 240 ng of infectious virus (control cells were incubated without virus), as measured by HIV-1 p24 antigen capture ELISA. Mixtures of target cells and virus were centrifuged for 2 h at 2000 RPM at 12 °C. Afterward, the viral inoculum was diluted 1:2 in R10 medium containing G418 1.5 mg/ml, and the cells and virus were placed into one well of a 12-well flat-bottom plate. The cells were then cultured adding fresh medium every 2 days. At 5 days post-infection, EGFP-CEM-NKr-CCR5-SNAP cells were harvested, washed twice with R10 medium, counted and divided into two tubes: one was stained for RFADCC as previously described; the second one was used to quantify the efficiency of infection and mAb binding. For intracellular p24 staining, the cells were washed once with PBS and stained with Live/Dead Fixable Near-IR Dead Cell Stain (Molecular Probes) and with 5 μl of (APC)-conjugated mouse anti-CD4 OKT4 mAb (eBioscience) for 30 min at RT. After a wash in wash buffer, the cells were then fixed and permeabilized using the Cytofix/Cytoperm Kit (BD-PharMingen, San Diego, Calif.) for 20 min at 4 °C. Subsequently, the permeabilized cells were washed once with the buffer provided by the manufacturer, resuspended and stained for 30 min at RT with 5 μl (PE)-conjugated mouse anti-p24 mAb (KC57-RD1; Beckman Coulter, Inc.). After two additional washes, HIV-1- or mock-infected PBMC was analyzed with an LSRII Fortessa flow cytometer (BD Biosciences) and data analysis was performed with FlowJo software (Tree Star, Inc., San Carlos, Calif.). Live and GFP positive cells were analyzed for intracellular p24-Ag and surface CD4.

### 2.5 HIV-1 cell–cell transmission in EGFP-CEM-NKr-CCR5-SNAP cells

Cell–cell virus transmission experiments were performed using CEM NKr CCR5 cells as virus-infected donor cells and EGFP-CEM-NKr-CCR5-SNAP cells as virus-acceptor targets. Accordingly, CEM NKr CCR5 cells were infected with HIV-1 Bal Molecular Clone and frozen at 3 days post-infection at the peak of infectivity (23% p24^+^ cells, Fig. 6 Panel C 0 h). The day before the co-culture experiment, the donor cells were thawed, rested overnight in R10 medium at 37°, 5% CO_2_ and then washed twice with PBS, keeping the supernatant as a control (Fig. 6 Panel A-Free virus Transwell-FVT). The infected CEM NKr CCR5 donors were plated with the acceptor EGFP-CEM-NKr-CCR5-SNAP targets at a ratio 1:1 (1 × 10^6^ donor: 1 × 10^6^ acceptor) for 7 days (Fig. 6 Panels A-C). For reference, the donor and acceptor cells were cultured at the same ratio in a separated trans-well chamber (12 mm Transwell® with 3.0 μm pore polycarbonate membrane insert (Corning #3402) to evaluate the ability of the donor-produced virus to infect the acceptor cells in a cell-free manner (Fig. 6 Panel A-Co-Culture Transwell-CCT). The readout of the newly infected targets was the count of GFP^+^ p24^+^ CD4^+^ cells over various time points. Every 24 h, an aliquot of the donor/acceptor cell combination was harvested and stained for the expression of surface CD4 and intracellular p24, as previously described in the previous section.

## 3 Results

### 3.1 Generation and characterization of the levels of CCR5 molecules on the EGFP-CEM-NKr-CCR5-SNAP cell line

**Fig. 1:**
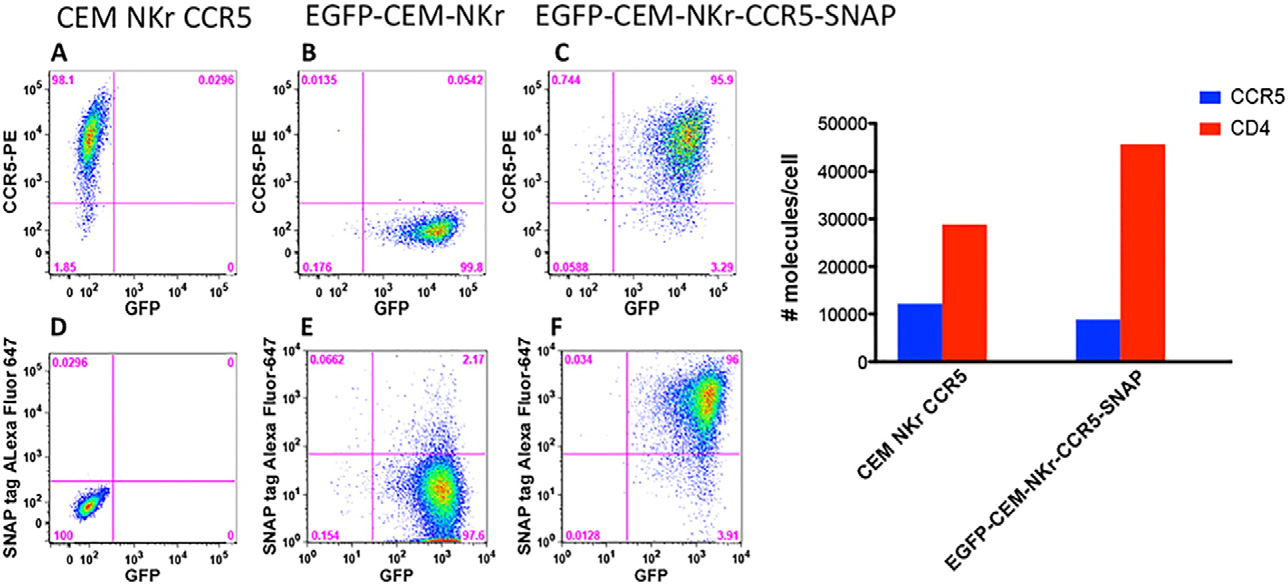
Generation and characterization of EGFP-CEM-NKr-CCR5-SNAP cells. Left panel. EGFP-CEM-NKr-CCR5-SNAP cells were generated by stable transfection of EGFP-CEM-NKr cells with Tag-lite pSNAP-CCR5 vector (pSNAP-CCR5, htfr). A–C. GFP and CCR5 expression in parental CEM-NKr-CCR5, EGFP-CEM-NKr and transfected EGFP-CEM-NKr-CCR5-SNAP cells. D–F. Labeling with surface SNAP tag-Alexa Fluor-647 dye vs. GFP expression in the three cell lines. Right panel. The levels of CCR5 and CD4 molecules expressed on EGFP-CEM-NKr-CCR5-SNAP cells in comparison to CEM NKr CCR5 were assessed by flow cytometry with Quantibrite PE calibration beads.

Keeping the original RFADCC assay format, we developed a more reliable and rapid approach to label the CEM target cells. To eliminate CSFE staining, we employed EGFP-CEM-NKr cells (Kantakamalakul et al., 2006) that constitutively express cytosolic GFP but are negative for CCR5 expression (Fig. 1, Panel A compared to Panel B). Then, we engineered the EGFP-CEM-NKr cells to stably express the fusion protein N-terminal SNAP-tagged CCR5 on the cell surface. After three rounds of sorting and limiting dilution culture, we successfully isolated a stable GFP^+^ clone that expressed CCR5-SNAP tag on the surface, as shown in Fig. 1, Panel C. Of note, the transfection of CCR5-SNAP did not affect the level of GFP expression (Fig. 1, compare panels B vs. C). More importantly, the CCR5-SNAP tag construct provided the advantage of using an anti-SNAP-tag (linked to CCR5) dye, which is relatively bright, non-toxic and specific for cell-surface (Fig. 1, Panel F), eliminating the background from non-CCR5-SNAP tag expressing cells (Fig. 1, Panel D and E). The SNAP tag provided the opportunity to use a second fluorochrome (SNAP-Surface Alexa Fluor 647) needed to identify the killed target cells in the RFADCC assay (Fig. 1, Panel F). We then determined the levels of CCR5 and CD4 molecules expressed on the cell surface with calibration beads and found that the new cell line expressed a physiologic level of CCR5 (Fig. 1, right panel blue bars). Additionally, we selected for cells with relatively high CD4 levels, which is typically useful for studying HIV-1 infection (Fig. 1, right panel red bars).

### 3.2 Optimized RFADCC assay with gp120-sensitized EGFP-CEM-NKr-CCR5-SNAP cells

**Fig. 2:**
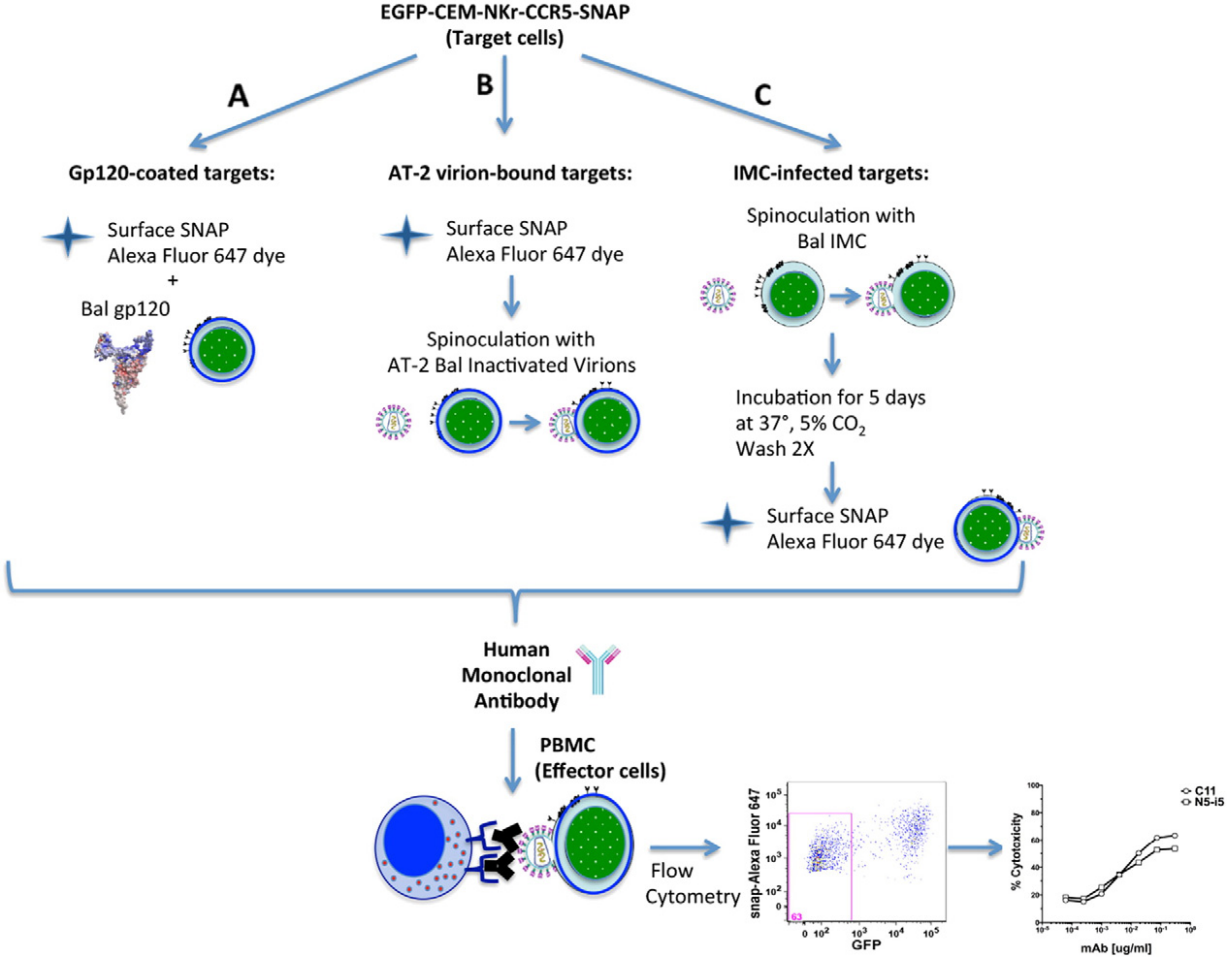
Schematic representation of the new RFADCC assay outline conducted with EGFP-CEM-NKr-CCR5-SNAP cells. The optimized assay was modified from Gomez-Roman et al. (2006). A. For gp120-based ADCC assay, EGFP-CEM-NKr-CCR5-SNAP cells were stained with SNAP-Surface Alexa Fluor 647 for 20 min at 37 °C with or without monomeric HIV-1 gp120. B. For AT-2 inactivated virus-based ADCC assay EGFP-CEM-NKr-CCR5-SNAP cells were stained with Alexa Fluor 647-SNAP tag dye first and then spinoculated with the inactivated virus. C. For IMC-infected targets-based ADCC assay, EGFP-CEM-NKr-CCR5-SNAP were spinoculated with IMC, cultured for 5 days, washed twice and then stained with Alexa Fluor 647-SNAP tag dye. Sensitized cells were incubated with dilutions of antibodies for 15 min at room temperature (RT) and subsequently with PBMC as effector cells for 2 or 3 h at 37 °C. Cells were then washed and fixed in 1% paraformaldehyde. The readout is the loss of GFP, as a direct measure of the percentage of target cells cytotoxicity mediated by the mAbs. After coating with monomeric HIV-1 Bal gp120 and adding PBMC as effector cells, we measured the ADCC activity of a reference HIV-1 mAb, C11. The cytotoxicity readout in the original RFADCC was measured as loss of CSFE, while in the modified RFADCC it was the loss of GFP.

To compare the original RFADCC with the modified RFADCC, CEM NKr CCR5 cells were stained with PKH26 and CSFE, while EGFP-CEM-NKr-CCR5-SNAP cells were stained with Alexa-Fluor 647 surface SNAP dye as summarized in Fig. 2.

**Fig. 3:**
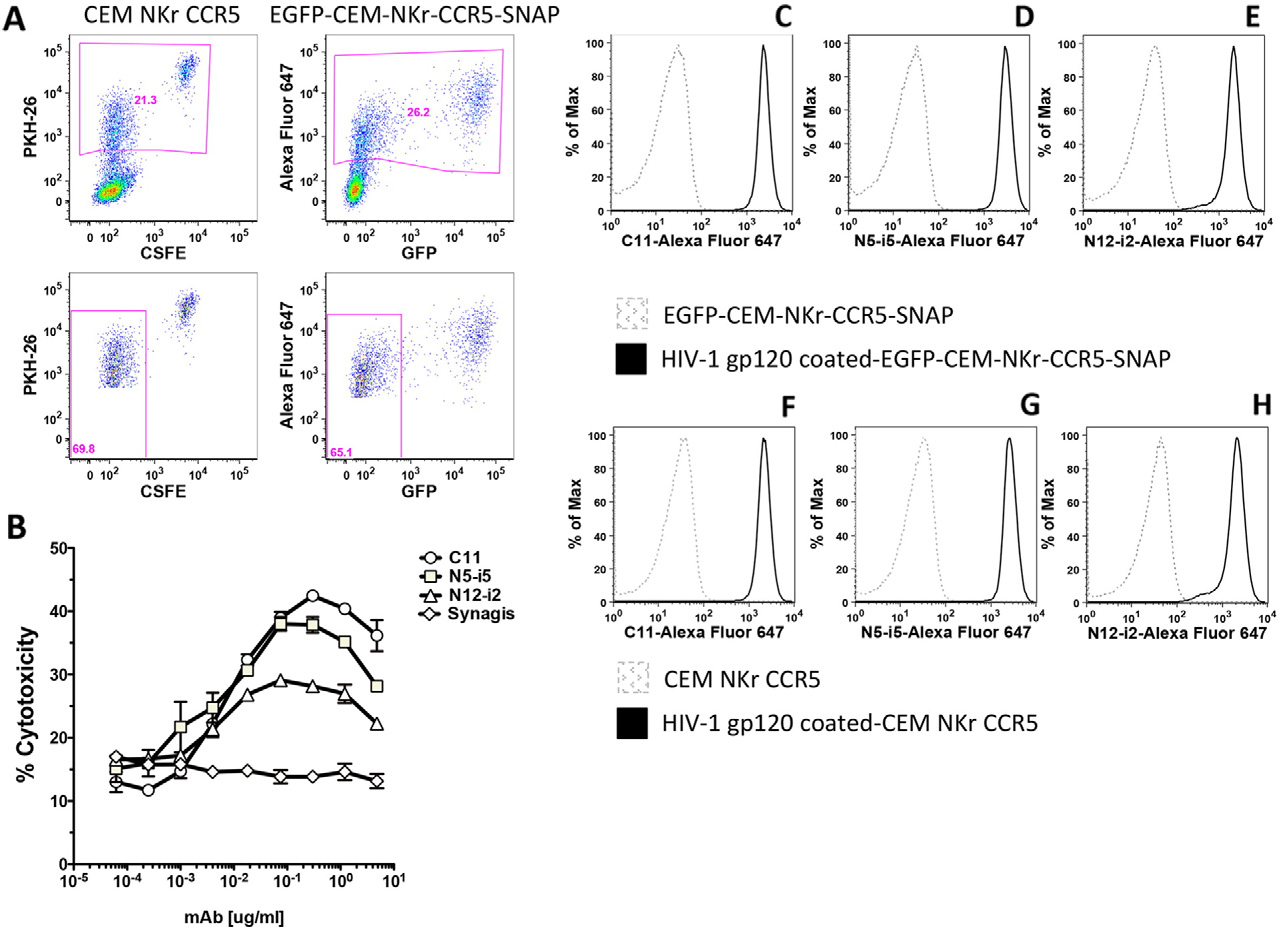
ADCC assay and mAbs surface staining conducted with cells coated with HIV-1 Bal gp120. A. Comparison of RFADCC assay layout with CEM NKr CCR5 vs. EGFP-CEM-NKr-CCR5-SNAP cells. CEM NKr CCR5 were stained with PKH-26 and CSFE (Gomez-Roman et al., 2006), while EGFP-CEM-NKr-CCR5-SNAP cells were stained with Alexa Fluor 647-SNAP tag dye. Killing by C11 mAb (1 μg/ml) is determined as loss of CSFE in PKH26-positive CEM NKr CCR5 cells or loss of GFP in SNAP-positive EGFP-CEM-NKr-CCR5-SNAP cells. B. Cytotoxicity curves for gp120-coated EGFP-CEM-NKr-CCR5-SNAP. The ADCC results are representative of three independent assays and the bars indicate the range of the values of cytotoxicity of duplicate samples. The binding of 1 μg/ml Alexa-Fluor 647-conjugated mAbs C11 (C and F), N5-i5 (D and G) or N12-i2 (E and H) was compared in gp120-coated-CEM NKr CCR5 vs. EGFP-CEM-NKr-CCR5-SNAP cells.

As shown in Fig. 3 Panel A, the two cell lines behaved similarly, having a comparable percentage of target cells migrating from the double stained population into the region containing the killed target cell population, positive only for PKH-26 or SNAP-Alexa Fluor 647 respectively. Subsequently, the new target cells were employed to measure the ADCC activity of two additional human anti-gp120 mAbs. As a negative control, we used an unrelated mAb, Synagis, specific for respiratory syncytial virus (RSV). The assay was performed by adding the double-labeled target cells to mAbs dilutions, allowing them to bind, and then adding PBMC as effectors. As shown in Fig. 3 Panel B, the new cellular system was able to detect the specific anti-gp120 ADCC activity of C11, N5-i5 and N12-i2 mAbs, with values comparable to the original RFADCC (Guan et al., 2013). As expected, the RSV mAb was negative in the induction of cytotoxicity. In addition, we evaluated the ability of the gp120-coated EGFP-CEM-NKr-CCR5-SNAP cell line as a tool to study the binding of the same mAbs. As shown in Fig. 3, we detected a very strong signal for all the Alexa Fluor 647-labeled antibodies (C11, N5-i5 and N12-i2), specific for the binding of gp120 molecules on EGFP-CEM-NKr-CCR5-SNAP cells (Panels C‐–E), compared to the negative control of uncoated cells (dashed greygray curve). Interestingly, the intensity of the binding detected in the new cell line was comparable to the standard gp120-coated CEM NKr CCR5 cells (Fig. 3 Panels F-H).

### 3.3 Optimized RFADCC assay with virion bound-EGFP-CEM-NKr-CCR5-SNAP cells

**Fig. 4:**
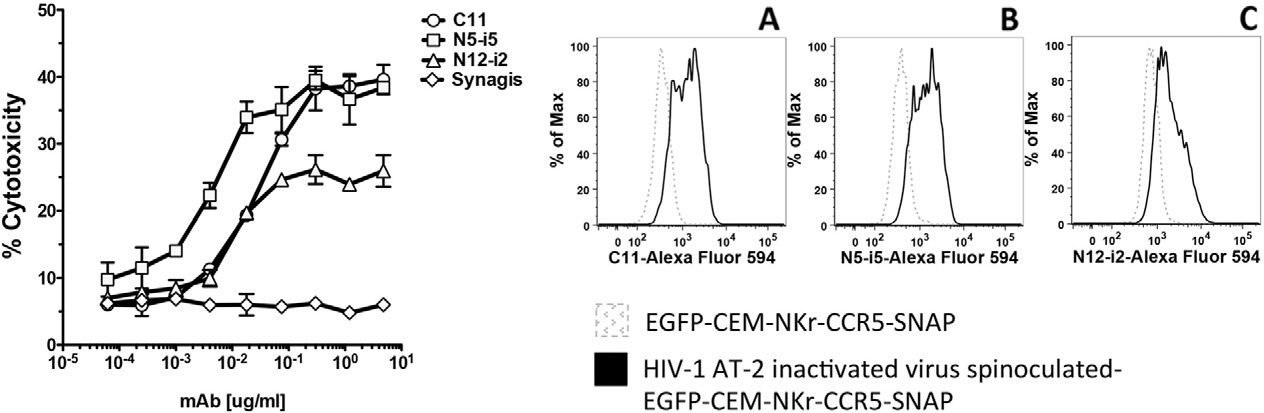
ADCC assay using cells spinoculated with AT-2 inactivated HIV-1 BaL virions. Left panel. EGFP-CEM-NKr-CCR5-SNAP cells were labeled with Alexa Fluor 647-SNAP tag and then spinoculated with HIV-1 Bal AT-2 virus at 2000 rpm for 2 h at 12 °C. After two washes, cells were incubated with dilutions of mAbs (C11, N5-i5, N12-i2 or Synagis) for 15 min at RT, then PBMC were added to the reaction for 3 h at 37 °C. At the end of the incubation, the samples were washed with PBS, fixed with 1% paraformaldehyde and analyzed by flow cytometry. The ADCC results are representative of three independent assays and the bars indicate the range of the values of cytotoxicity of duplicate samples. Right panel. The efficiency of the spinoculation was evaluated by cell surface staining with 2 μg/ml Alexa Fluor-594-conjugated mAbs C11 (Panel A), N5-i5 (Panel B) or N12-i2 (panel C).

In order to evaluate the possibility of using the new cell line to identify mAbs that mediate ADCC against cell-bound intact HIV, we spinoculated the EGFP-CEM NKr CCR5-snap cells with AT-2 inactivated HIV-1 Bal, as described in the Materials and Methods section (Rossio et al., 1998, O'Doherty et al., 2000). This approach allows the evaluation of immunogenicity of envelope epitopes exposed upon binding of cell surface CD4. As shown in Fig. 4 (left panel) in the modified system, the reference antibodies were able to induce strong cytotoxicity against cell-bound virus. The ADCC values were comparable to those obtained in the original RFADCC with CEM NKr CCR5 cells bound with the same virus (Guan et al., 2013). Moreover, we quantified the efficiency of the spinoculation-mediated virus binding by testing binding with the reference mAbs labeled with Alexa Fluor-594. We were able to detect a strong signal for all the antibodies tested (Fig. 4 right Panels A-C). Of note for the virus-bound cells, the values for ADCC activity and binding ability were lower than with the gp120-coated cells because of the relatively low number of envelope spikes per virus and the low numbers of cells that can be bound synchronously by the virus in vitro (compare Fig. 3 [gp120] vs Fig. 4 [virus bound]).

### 3.4 Infection and optimized RFADCC assay with EGFP-CEM-NKr-CCR5-SNAP cells

**Fig. 5:**
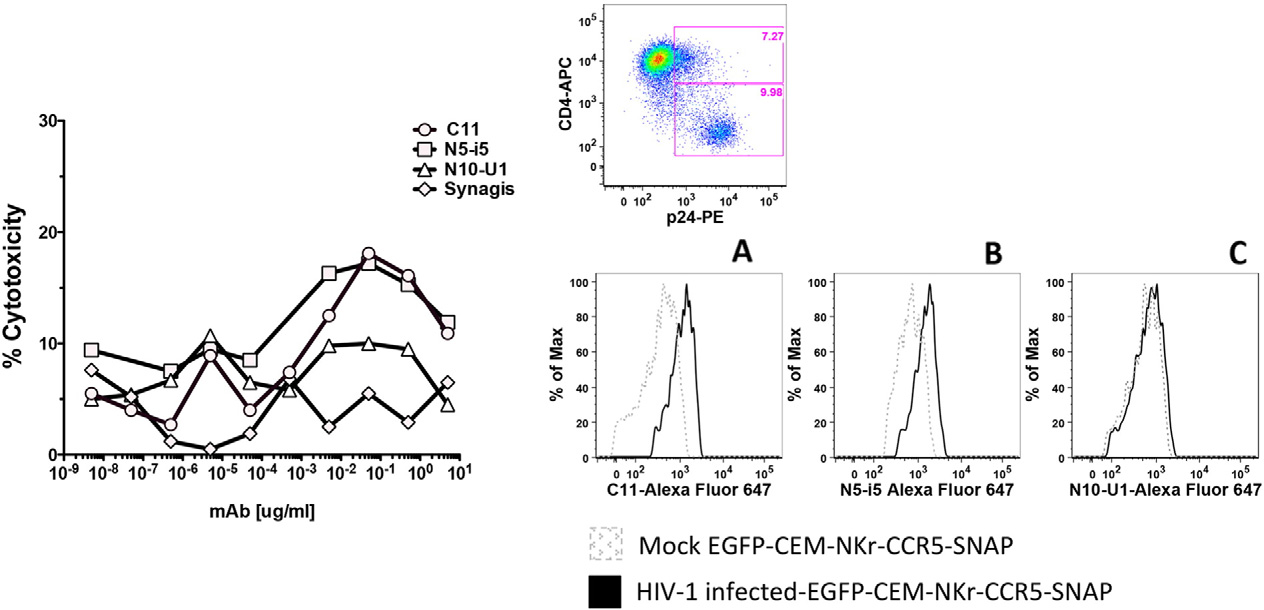
ADCC assay using cells infected with HIV-1 BaL IMC. Left panel. EGFP-CEM-NKr-CCR5-SNAP cells were spinoculated with HIV-1 Bal molecular clone at 2000 rpm for 2 h at 12 °C. After 5 days of co-culture with the virus, cells were washed twice, labeled with Alexa Fluor 647-SNAP tag dye and incubated with dilutions of mAbs (C11, N5-i5, N10-U1 or Synagis) for 15 min at RT, then PBMC were added to the reaction for 3 h at 37 °C. At the end of the incubation, the samples were washed with PBS, fixed with 1% paraformaldehyde and analyzed by flow cytometry. The ADCC data represent the typical results obtained in three independent experiments. Upper right panel. The efficiency of the infection was evaluated by staining of the cells with live/dead (not shown), CD4-APC and p24-PE. Lower right panel. Binding of infected EGFP-CEM-NKr-CCR5-SNAP cells with 5 μg/ml Alexa Fluor-647-conjugated mAbs C11 (panel A), N5-i5 (panel B) or N10U1 (panel C).

In order to determine whether the modified cell line was suitable for virus infection and thus use in an infected-cell RFADCC assay, we spinoculated the EGFP-CEM-NKr-CCR5-SNAP cells with HIV-1 subtype B Bal infectious molecular clone (IMC), as previously described (Alpert et al., 2012). At 5 days post-infection Bal-spinoculated EGFP-CEM-NKr-CCR5-SNAP cells presented a high cellular viability (approximately, 90%), comparable to control uninfected cells (data not shown). The rate of infection was about 20% (as shown by the presence of p24 in Fig. 5 right upper panel). As expected, a high percentage of p24^+^ cells down-regulated the surface expression of the CD4 molecule (Veillette et al., 2014). We employed the infected cells as targets for our high throughput RFADCC. Interestingly, we were able to detect binding and cytotoxicity of the CD4i reference mAbs C11 and N5-i5, the epitopes of which are normally masked in an unbound, native Env trimer (Acharya et al., 2014, Veillette et al., 2014) (Fig. 5 left panel and right panels A-B); this is most likely due to the residual surface membrane CD4 expression in a fraction of p24^+^ infected cells, rendering them sensitive to ADCC with these CD4i mAbs (Veillette et al., 2014). In addition, we revealed low level binding and ADCC activity for N10-U1, which is specific for the immunodominant domain of gp41 (Fig. 5 left panel and right Panel C). As expected, Synagis was negative for the ADCC activity and for binding to Bal infected EGFP-CEM-NKr-CCR5-SNAP cells (data not shown). This result constitutes additional evidence that the chimeric CCR5-SNAP tag is able to act as a functional co-receptor for HIV-1 during virus entry and fusion with the cellular membrane.

### 3.5 A new tool to determine cell–cell virus spread

**Fig. 6:**
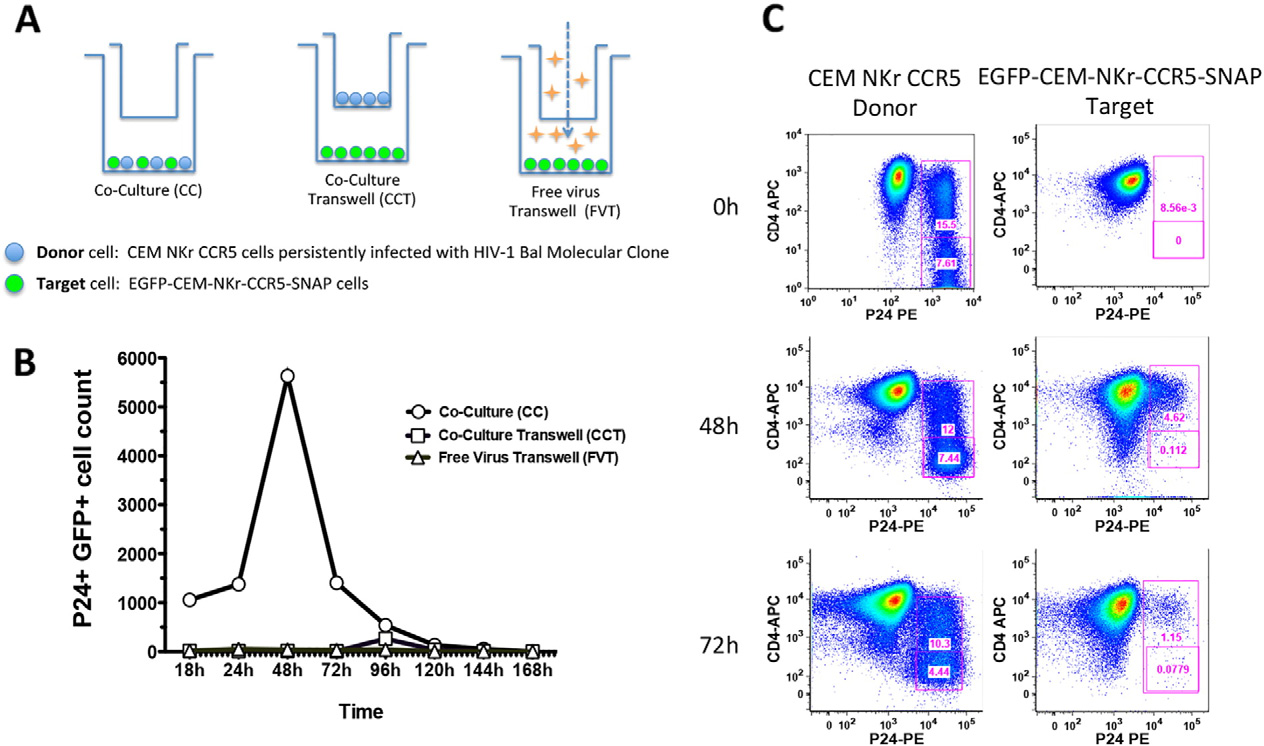
EGFP-CEM-NKr-CCR5-SNAP cells: New tool to study the cell–cell virus spread. A. Schematic representation of the experimental design (modified from Zhong et al., 2013). CEM NKr CCR5 cells, infected with HIV-1 Bal molecular clone, were used at the peak of infection as virus donors. EGFP-CEM-NKr-CCR5-SNAP cells were used as virus acceptor targets of the infection transmitted cell to cell. Donors and targets were plated together to allow the spread of the virus, and the readout of the newly infected targets was the count of GFP^+^/P24^+^ cells. Background of cell-free virus spread was determined with donors and targets separated in Transwell chambers and with targets subjected to supernatants harvested from infected donor cells. B. and C. The count of GFP^+^/p24^+^/CD4^+^ or ^−^ cells was monitored for 7 days from the beginning of the co-culture by flow cytometry.

HIV-1 has been shown to be capable of two modes of propagation: direct infection by cell-free virions and cell-to-cell transmission (Sattentau, 2008). Notably, cell-to-cell spread from infected to non-infected cells occurs through the formation of virological synapses (McDonald et al., 2003, Sattentau, 2008, Hubner et al., 2009) and has been shown to be a more rapid and efficient mechanism compared to free virus-cell diffusion. This feature supports the hypothesis that cell–cell transmission may be a relevant mode of virus dissemination in infected individuals (Dimitrov et al., 1993, Carr et al., 1999, Chen et al., 2007, Sourisseau et al., 2007). Thus, it is important to have a tool that allows the methodical evaluation of mAbs or other molecules compatible with this type of infection. Accordingly, we designed an experimental method based on Zhong et al's. (2013) system in which the authors compared the efficiency of HIV-1 transmission by cell-free virus vs. cell–cell transmission in co-cultures. To evaluate it with our EGFP-CEM-NKr-CCR5-SNAP cell line, we persistently infected CEM NKr CCR5 cells with HIV-1 Bal molecular clone, using these cells as donors of replication-competent virus at the peak of the infection, as found by the levels of p24 expression (Fig. 6, Panel C, Donor-0 h). With the EGFP-CEM-NKr-CCR5-SNAP cells as the virus infectable targets, these cells were directly co-cultured with the virus infected donors or separated from the donors by a trans-well membrane for 168 h. An additional control was the supernatant harvested from the infected CEM NKr CCR5 cells just before the experiment (summarized in scheme Fig. 6, Panel A). The readout of the assay was the count of GFP^+^/p24^+^ cells, representing newly infected target cells. As shown in Fig. 6, Panel B, the co-culture of infected donors with uninfected targets allowed the transmission of the virus through cell–cell contacts, reaching a peak of GFP^+^/p24^+^ cell count at 48 h. Additionally, flow-cytometry analysis allowed the characterization of the levels of expression of CD4 on the surface of the newly infected GFP^+^/p24^+^ cells. In Fig. 6, Panel C, we show that by 48 h of co-culture, levels of CD4 on the cell surface had decreased from 100% to 5% and by 72 h, CD4 levels were further decreased to 1%. Thus, using this culture system, the physical contact of cells was much more efficient in spreading the infection (Fig. 6, Panel B), than culturing uninfected cells with cells actively producing virus or with cell-free virus-containing supernatant, as shown in Fig. 6, Panel B.

## 4 Discussion

The RFADCC assay has provided an important contribution in the characterization of antibodies that mediate strong cytotoxicity against HIV-1 sensitized cells. Here we describe an optimization of the original RFADCC that has been modified in the following ways: to replace the time consuming staining of target cells with two dyes and subsequent multiple washings, we took advantage of the cell line EGFP-CEM-NKr (Kantakamalakul et al., 2006) that constitutively expresses one of the two fluorochromes needed to identify target cells. Next, to include a second fluorochrome and replace the PKH26 dye, which is relatively toxic, we transfected cells with a SNAP tag-linked CCR5, and then stained the targets with an Alexa Fluor dye directed to the SNAP tag. The first part of the ADCC procedure now is dramatically faster and more reproducible (summarized in Fig. 2). Moreover, as we show in Fig. 3, Fig. 4, the results obtained both in gp120-coated or AT-2 inactivated virus bound-cells are very comparable to each other and consistent with what we had previously reported (Guan et al., 2013). We also demonstrated that the new cell line can serve as a susceptible target for HIV-1 infection and subsequently be employed as targets for ADCC activity mediated by anti-HIV-1 antibodies (Fig. 5). These results are significant for high-throughput evaluation of antibody responses to epitopes exposed earlier during viral entry and later during viral budding post-infection. Finally, we assessed the possibility of utilizing EGFP-CEM-NKr-CCR5-SNAP cells to evaluate cell–cell spreading of HIV-1, which has been demonstrated to be the most effective for virus transmission. In this model, we exploited the expression of the GFP to identify the modified target cells that have been newly infected during the experiment (Fig. 6).

Taken together, the use of the EGFP-CEM-NKr-CCR5-SNAP cell line makes the RFADCC a faster and more reliable tool for the evaluation of the cytotoxicity against cells infected with many human and non-human pathogens, such as HIV-1 and SIV viruses.
